# Household Air Pollution and High Blood Pressure: A Secondary Analysis of the 2016 Albania Demographic Health and Survey Dataset

**DOI:** 10.3390/ijerph19052611

**Published:** 2022-02-24

**Authors:** Mustapha S. Abba, Chidozie U. Nduka, Seun Anjorin, Olalekan A. Uthman

**Affiliations:** 1 Division of Health Sciences, Warwick Medical School, The University of Warwick, Coventry CV4 7AL, UK; c.nduka@warwick.ac.uk (C.U.N.); seun.anjorin@warwick.ac.uk (S.A.); 2 Warwick Centre for Global Health, Division of Health Sciences, University of Warwick Medical School, Coventry CV4 7AL, UK; olalekan.uthman@warwick.ac.uk; 3 Division of Epidemiology and Biostatistics, Department of Global Health, Faculty of Health Sciences, Stellenbosch University, Francie van Zijl Drive, Tygerberg, Cape Town 7505, South Africa

**Keywords:** hypertension, high blood pressure, household air pollution, polluting fuels, Albania, low- and middle-income countries

## Abstract

Background: Hypertension is a significant public health problem in low- and middle-income countries (LMICs). This study aimed to examine the association between household air pollution (HAP) and blood pressure using data from the 2016 Albania Demographic Health and Survey (DHS). Methods: We computed the odds ratio (OR) for the prevalence of hypertension between respondents exposed to clean fuels (e.g., electricity, liquid petroleum gas, natural gas, and biogas) and respondents exposed to polluting fuel (e.g., kerosene, coal/lignite, charcoal, wood, straw/shrubs/grass, and animal dung). Result: The results show that participants exposed to household polluting fuels in Albania were 17% more likely to develop hypertension than those not exposed to household air pollution (OR = 1.17, 95% CI 1.10 to 1.24). Subgroup analysis revealed that the odds of hypertension were more significant among women (OR = 1.22, 95% CI 1.13 to 1.31), rural residents (OR = 1.12, 95% CI 1.04 to 1.22), and participants aged >24 years (OR = 1.35, 95% CI 1.12 to 1.62) who were exposed to household polluting fuels compared to their counterparts who were not exposed. In summary, the results of the study show significant associations between household air pollution and hypertension risk overall, especially among women, rural dwellers, and people aged >24 years in Albania. Conclusion: In this study, an association between household air pollution and the risk of hypertension was found, particularly among low-income households, those with no education, women, and those who live in rural areas.

## 1. Introduction

High blood pressure remains a public health problem in low- and middle-income countries (LMICs). It is a significant risk factor for cardiovascular disorders, including stroke and coronary heart disease [1,2,3,4].

In 2015, hypertension affected 1.13 billion individuals worldwide and resulted in 9.4 million deaths [5,6]. By 2025, the prevalence is projected to increase by 30% according to the World Health Organization [7]. Hypertension is projected to increase with growing population density and ageing in males and females [8,9]. The prevalence is high in Albania, especially among older adults; it is the primary driver of death and disability [10]. The environment is known to influence an individual’s health outcome; for example, a good and secure environment will improve one’s quality of life [7,11]. However, as a result of globalisation and industrialisation, the environment/home is currently a source of air pollutants that significantly affect the health and well-being of individuals, particularly in low- and middle-income countries [7,12]. Notably, the automotive industry and the establishment of factories have contributed to air pollution, especially in cities where most of the population reside. This development influenced the emergence of many diseases that pose high risks to life [12]. Therefore, protecting the environment and the air is the responsibility of every individual.

Clean air is considered one of the essential human health and well-being requirements [13]. However, in 2016, more than 90% of the world population was exposed to air quality levels that exceeded the World Health Organization (WHO) Air Quality Guideline limits [14]. Air pollution is the fourth most significant overall risk factor for human health globally, following high blood pressure, dietary risks, and smoking [15]. It has been associated with three of the leading causes of death worldwide, with significant shares in air-pollution-related mortality: stroke (26%), ischemic heart disease (20.2%), and primary cancer of the trachea, bronchus, and lung (19%) [16,17]. Air pollution has been linked to 7 million deaths globally and more than 100 million disability-adjusted life years (DALYs) annually [18].

Household air pollution is mainly generated by the utilisation of inefficient household combustion for cooking, heating, and lighting solid biomass (e.g., wood, cow dung, charcoal, and crop residues), which is common in most LMICs, including Albania [19]. An elevated level of fine particulate matter (PM 2.5) is an emerging hypertension risk factor [20,21]. House air pollutants such as carbon monoxide (CO) and particulate matter (PM) are classified to be hazardous, resulting in morbidity and mortality [22]. The combustion of solid fuels increases the risk of developing cardiovascular diseases, including hypertension and other heart diseases, especially in LMICs [23,24]. In 2010, household air pollution from solid fuels accounted for 3.5 million deaths and 4.3% of global disability-adjusted life years [21]. An estimated 3 billion people are exposed to household air pollution (HAP) from cooking with solid fuels, and most deaths result from using bad fuels, especially in LMICs [25,26]. The inhalation of several air pollutants causes acute autonomic imbalance and promotes the release of a variety of pro-oxidative, inflammatory, and haemodynamically active mediators into systemic circulation [21]. Consequently, numerous adverse responses, including elevations in blood pressure (BP), can instigate other cardiovascular-related disorders such as myocardial infarction, stroke, heart failure exacerbation, arrhythmia, and cardiovascular-related death [22,26,27]. Higher PM and BP levels are individually linked to premature morbidity and mortality. The biological interconnection between these two risk factors and air pollution causes a significant threat to global public health [21]. 

High blood pressure and air pollution were found to be significant risk factors that cause the most deaths and disabilities in Albania. At the same time, ischemic heart disease and stroke remain the top causes of death, according to the Institute of Health Metrics and Evaluation (IHME) report [28]. Socioeconomic changes in the early 1990s resulted in increasing air pollution in Albania [21]. 

Although many efforts have been made to promote the use of clean fuels, nearly 2.7 billion people lack access to clean cooking facilities, relying on biomass, coal, or kerosene to meet their most basic energy needs [29]. The percentage of the population with access to clean fuels and technologies is increasing in most parts of the world. However, the rate of increase is not commensurate with population growth in most low- and middle-income countries, which potentially suggests there will be a rise in the prevalence of hypertension in most low- and middle-income countries [14]. Available evidence demonstrates the impact of ambient air pollution and its association with cardiovascular diseases, which has been well studied in high-income countries with consistent findings [30,31] However, indoor air pollution is an emerging risk factor for cardiovascular disease in LMICs, including Albania [32]. The aetiology of hypertension and its associations with indoor pollution is not well researched. Therefore, this study aims to provide additional evidence and new insights into new preventive measures that will reduce the impact of indoor air pollution in resource-limited settings.

## 2. Methods

### 2.1. Study Design and Sampling Technique

This study was based on the secondary analyses of cross-sectional, population-based data from the 2018 Albania Demographic and Health Survey (DHS). The survey was conducted to collect data on demographic, environmental, socioeconomic, and health issues (family planning, infertility, immunisation, and the nutritional and health status of children, mothers, and fathers). The sample for the 2018 Albania DHS was nationally representative and covered the entire population in the country. The data collection procedures used have been published elsewhere. Data were collected by conducting face-to-face interviews with women and men who met the eligibility criteria. This study is based on an analysis of existing survey data with all identifier information removed. All study participants gave informed consent before participation, and all data were collected confidentially.

This analysis used cross-sectional data from the most recent 2018 Albania DHS. DHSs are nationally representative household surveys that provide information on a wide range of monitoring and impact evaluation indicators in a population, including nutrition, health, and other vital characteristics. The surveys have large sample sizes (usually between 5000 and 30,000 households). A DHS is conducted approximately every five years to allow for comparisons to be made over time. 

In a DHS, the sample is generally representative at the national, residential (urban or rural), and regional levels. The survey utilises a stratified two-stage cluster design. Enumeration areas (EAs) are drawn from a census file during the first stage. In each EA selected, the second stage involves drawing a sample of households from an updated list of households. The national statistical office typically implements a DHS in a country, with data collection lasting between 5 and 6 months.

### 2.2. Outcome Variable

This study’s primary outcome was hypertension, which included respondents who had a systolic blood pressure >140 mmHg, diastolic blood pressure >90 mmHg, or who were taking anti-hypertensive drugs at the time of the survey. Blood pressure was measured three times in 10-min intervals with small, medium, and large cuff sizes depending on the cuff size of the respondent. The average of the second and third measurements was used to categorise hypertension [33].

### 2.3. Exposure Variable

Exposure to household air pollution was grouped into two categories in this analysis based on exposure to cooking smoke: “clean fuels”, including electricity, liquid petroleum gas (LPG), natural gas, and biogas, and “polluting fuels”, including kerosene, coal/lignite, charcoal, wood, straw/shrubs/grass, and animal dung. 

### 2.4. Control Variables

The following individual-level factors were included in the study: sex of the respondent (male versus female), respondents’ age in years, educational attainment (no education, primary school, secondary school, or higher); marital status (never married, currently married, or ever married), occupation (working versus not working), cigarette smoking (yes versus no), health insurance (yes versus no), problems getting the money needed for treatment (yes versus no), media access (radio, television, or magazine), and indoor air pollution (cooking fuel type: low-pollution fuel or high-pollution fuel). Weight was measured to the nearest 0.5 kg with subjects in light clothing, and height was measured to the nearest 0.1 cm. Body mass index (BMI) was calculated and categorized as underweight (<18.5 kg/m^2^), overweight (25–29.9 kg/m^2^), and obese (≥30 kg/m^2^). The DHS did not collect direct information on household income and expenditure. We used the DHS wealth index as a proxy indicator for the socioeconomic position of the participants. 

### 2.5. Statistical Analysis

The analytical approach included descriptive as well as bivariable and multivariable analyses. The descriptive statistics show the distribution of respondents by the key variables. Values are expressed as absolute numbers (percentages) and means (standard deviation) for categorical and continuous variables. Individual weights were used for descriptive statistics in this study. We computed the odds ratio (OR) for the prevalence of hypertension between respondents exposed to clean fuels and respondents exposed to polluting fuel. An OR more significant than 1 suggested hypertension prevalence estimates were more prevalent among respondents exposed to polluting fuels. Conversely, a value less than 1 indicated that the hypertension prevalence estimates were more prevalent among respondents exposed to/using bad/polluting fuels, in addition to estimating the association between indoor air pollution and hypertension for all participants. The association was stratified by age, sex, educational attainment, occupation, body mass index category, and the place of residence of the participants. The life course association between indoor air pollution and hypertension was equally examined.

Next, we used bivariable analyses to examine the association between each variable and the dependent variable of high blood pressure. Contingency tables were analysed using the Pearson χ^2^ test or Fisher exact test. Multivariable logistic regression analyses were used to examine the net effects of the explanatory variables on the dependent variables. For multivariate analyses, household air pollution and other control variables listed above that were significant in the bivariable study (*p* < 0.05) were included in the regression model in a single block to control for possible confounding factors between them. The magnitude and direction of association were expressed in the adjusted odds ratios, and significant levels were defined as *p*-values. Regression diagnostics were used to judge the goodness-of-fit of the model. They included the tolerance test for multicollinearity, its reciprocal variance inflation factors (VIFs), the presence of outliers, and estimates of adjusted R square of the regression model. In addition, the Hosmer–Lemeshow goodness-of-fit test was used. None of the results of the tests provided any cause for concern. Thus, the models provided robust and valid results. The significance tests were two-tailed, and statistical significance was defined at the alpha level of 0.05. Stata 17 was used for all analyses.

## 3. Results

### 3.1. Sample Characteristics

The characteristics of the participants are summarised in Table 1. The prevalence of hypertension was 29.9%, and the number of respondents with blood pressure sampled in Albania included in the analysis was 20,846. Many of the respondents were females (71.3%); in comparison, 28.9% of the respondents were males. More than half of the respondents had secondary or higher education (53%), while 47% either had no education or primary school education only. The majority of the respondents belonged to the 45–54 age group (25%). In comparison, 13% of the respondents belonged to the 55–64 age group. About 28% of the respondents in Albania belonged to the poorest wealth quintile, while 11% belonged to the wealthiest quintile. A total of 59.1% of the respondents were not working, while 40.8% reported that they were working. Approximately 40% of the respondents had a normal weight, while 57% were either overweight or obese. About 54% of the participants lived in rural areas, while 45% lived in urban areas. Respondents exposed to household air pollution (bad fuel) in Albania were 16.5% more likely to develop hypertension than respondents not exposed to house air pollution or respondents who used clean fuel.

The percentage of individuals with hypertension was higher among females exposed to/using bad fuel than males (20.8% versus 9%, *p* < 0.0001). Respondents with primary school education or no education were more likely to be exposed to indoor air pollution. The prevalence of hypertension was significantly higher in those with no education or primary school education than respondents with higher education (16.9% versus 12.9%). The prevalence of hypertension was significantly higher among the older age groups of 35 years and above. Approximately 12% of the 45–54-year-old respondents were reported to be hypertensive, while the prevalence was lowest among the age group of 15–24-year-olds (1.4%). The level of hypertension was higher among the poorest households than among the wealthiest households (9.2% versus 3.0%, *p* < 0.0001). The prevalence of hypertension was higher among respondents who were not working (17.0%) than respondents who did work (12.0%). Respondents who were overweight (11.8%) and obese (11.5%) were significantly more likely to be hypertensive than those who had a normal weight (6.4%) and were underweight (0.18% *p* < 0.0001). 

### 3.2. Association between HAP and Hypertension

**Figure 1:**
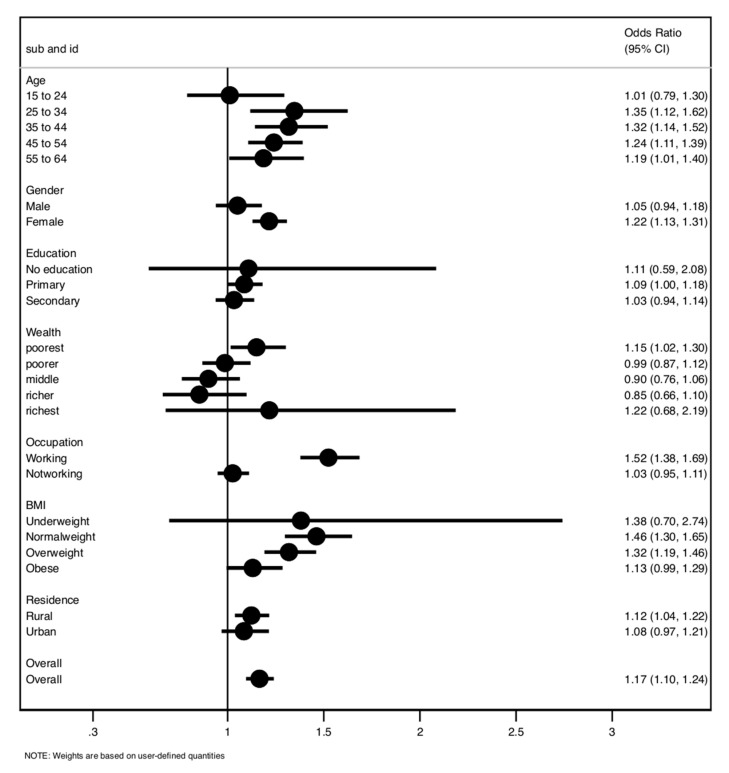
Association between exposure to HAP and hypertension risk.

The measure of association between house air pollution and hypertension is summarised in Figure 1 and Table 2. In the adjusted analysis, participants exposed to household air pollution were 17% more likely to have developed hypertension compared to those not exposed to indoor air pollution (OR = 1.17, 95% CI 1.10 to 1.24), such that 2310 of the 4906 (32.0%) individuals exposed to household air pollution had hypertension; in comparison, 3917 of the 9699 (28.8%) individuals not exposed to HAP developed hypertension. When stratified by the sex of the participants, the association was only significant among women, such that women exposed to HAP were 22% more likely to have developed hypertension than women not exposed to HAP (OR = 1.22, 95% CI 1.13 to 1.31). In addition, the association was only significant among rural residents. Rural residents exposed to HAP were 12% more likely to develop hypertension than those not exposed to HAP (OR = 1.12, 95% CI 1.04 to 1.22). The association was more significant among participants aged 25 to 54 years old than among those aged 15 to 24 years old. 

After controlling for other potential confounding variables in the adjusted analyses, the association between hypertension and household air pollution remained statistically significant (OR = 1.09, 95% CI 1.01 to 1.19). The following traditional risk factors remained statistically significant: women were 20% less likely to be hypertensive (OR = 0.80, 95% CI 0.74 to 0.86); participants with primary school education were 21% more likely to be hypertensive (OR = 1.21, 95% CI 0.12 to 1.30) than those with secondary school or higher education; overweight and obese respondents were more likely to be hypertensive; and the odds of hypertension increased with increasing age.

## 4. Discussion

The dataset used for this analysis was nationally representative population data from Albania, which we used to examine the association between indoor air pollution exposure in low- and middle-income countries. In Albania, the prevalence of developing hypertension was significantly higher among respondents exposed to house indoor air pollution. The odds of developing hypertension increased by 17% among respondents exposed to indoor air pollution than respondents not exposed. This study revealed that exposure to cooking solid fuels was significantly associated with increased blood pressure levels. Biomass fuel for cooking was associated with greater hypertension risk than the use of cleaner-burning LPG [11,27,34]. A study conducted by Li et al. found a positive association between increased indoor air pollution and solid fuel use and hypertension risk [34]. This could be due to high dust contents originating from local natural sources and metallurgical combinations [21]. An assessment revealed that dust levels in streets and in houses was approximately at the same level as those on roads; as such, more significant amounts of PM are emitted both at home and on the streets [35]. Conversely, Ofori et al. and Fatmi et al. found a negative association between indoor air pollution and hypertension; notably, this inconsistency might have been due to the type of fuel, location, and other confounding factors [36,37]. 

The study demonstrated that the percentage of individuals with hypertension was higher among females exposed to household air pollution than males and females who used clean fuels for cooking. This finding is consistent with the study carried out by Arku and colleagues [25]. The result indicated a significant increase in blood pressure among women cooking with solid fuels, with substantially high blood pressure among rural women. Supporting this finding, Gordon et al. stated that women have a leading role in domestic cooking in more cultures than men do [19]. Balmes also found more substantial effect estimates for women due to intense daily cooking smoke exposure [38]. 

Socioeconomic status is a significant predictor of exposure to HAP in most cultures. In any context, less expensive fuel options are generally less efficient fuels, produce more household air pollution, and are used by people with the most poorly designed homes. In this study, the results demonstrate variations in population exposure to indoor air pollution across socioeconomic groups. Respondents with primary school or no education had a significantly higher prevalence of hypertension than respondents with higher education. Additionally, the prevalence of hypertension was higher among respondents in the poorest households than those from the wealthiest households. Supporting this finding, Apte and Salvi reported that the poor socioeconomic strata of society in rural settings live in poorly ventilated houses and use bad fuel as a source of energy, which contributes immensely to hypertension risk [11]. The situation is aggravated by inappropriate infrastructure such as electricity in Albania, making it difficult to use electricity as a source of energy. In contrast, houses in developed countries use clean liquefied petroleum gas, natural gas, or electricity for cooking. Many of these houses are well ventilated and also have green areas and fitted air conditioners. 

The prevalence of hypertension was more significant among respondents not working than respondents who worked in Albania. Most workers live in the cities, can afford clean fuel as their energy source, and access good health care. Indoor air pollution has the most significant impact upon vulnerable populations within society due to limited resources to afford clean sources of energy and exposure due to underlying health conditions [39]. The prevalence of hypertension was significantly higher among the older age groups aged 35 and above. Giorgini et al. classified the elderly as vulnerable subjects with slightly higher blood pressure than younger groups following exposure to indoor air pollution [21]. Similarly, Deng et al. observed that older people exposed to biomass are more likely to develop hypertension in China [27].

The prevalence was significant among rural residents. Rural residents exposed to HAP were more likely to develop hypertension than those not exposed to HAP. Place of residence has been associated with HAP. Most of the population in LMICs live in rural and poor urban areas and mainly use biomass fuel as a source of their energy [40]. The prolonged inhalation of smoke from biomass cooking can increase the prevalence of hypertension [21].

### 4.1. Strengths and Limitations

This study used nationally representative data to evaluate the association between indoor air pollution and hypertension risk in a resource-limited setting. Despite the strengths of this study, we consider a number of limitations. For instance, more than 70% of the respondents were women, which may explain why the association between HAP and hypertension was significant among women but not men. 

In addition, we could not account for certain factors that might affect actual indoor air pollution and blood pressure measurement, including information on cooking time and the type of building and ventilation, which was shown to be linked to the prevalence of hypertension. The DHS data do not include information on other or secondary cooking fuels in the household. Some households reported using more than one fuel, which would lead to misclassification in the exposure definition and bias the estimated associations [41] The study did not include information on ambient air pollution, which may also be associated with high blood pressure. There was little or no information on the use of blood pressure medication. Lastly, several studies have used self-reported primary cooking fuel as a practical proxy for HAP; this is an inherently limiting indicator [42]. For cooking, heating, and lighting, one-third of the world’s population uses organic materials such as wood, dung, or charcoal (biomass fuel). This type of energy use is linked to high levels of indoor air pollution, an increase in the incidence of respiratory infections such as pneumonia, tuberculosis, and chronic obstructive pulmonary disease, low birth weight, cataracts, cardiovascular events, and all-cause mortality in both adults and children [43]. The mechanics underlying these connections are not completely known. Much of the health-related exposure to air pollution from cooking fuel happens outside of homes, not simply within them [44]. Solid cooking fuel is toxic enough to have a significant impact on ambient (outside) air pollution levels and, as a result, can induce illness far away from the source [44]. Carbon monoxide, formaldehyde, and other hazardous chemicals released by natural gas and propane burners can be toxic to people and pets. Cooking with a wood stove or fireplace can produce a lot of wood smoke, which can pollute both indoor and outdoor air [45].

### 4.2. Policy Implications

Indoor air pollution and the use of cooking fuels can be controlled by introducing clean fuel/electricity such as liquid petroleum gas (LPG), which is a fuel recognised to reduce HAP levels. Governments should adopt renewable energy incentives to reduce dependence on solid fuels [46]. The findings will inform future intervention studies and policy changes by generating knowledge to effectively control high blood pressure in low- and middle-income countries.

## 5. Conclusions

The study found an association between household air pollution and the risk of hypertension, particularly among low-income households, those with no education, women, and those who live in rural areas. The findings suggest that the risk of hypertension may be reduced by eliminating or reducing indoor air pollution, particularly among Albania’s most vulnerable groups and those groups in other similar countries in resource-limited settings. One of the most significant contributions to residential air pollution is the use of bad fuels for cooking and heating. Therefore, to minimise house air pollution, it is imperative to create awareness about household air pollution and its health effects to reduce the risk of cardiovascular disease in the population at large. Major interventional strategies need to be implemented at various levels to reduce household air pollution and its effects on health. Research and consolidating nationwide data from all countries will help to generate regional data regarding household air pollution in different regions to inform policy and decision making through behavioural interventions and cost-effective methods to improve the types of fuels used for cooking and heating. We have merely defined household air pollution as the self-reported exposure to ‘polluting fuels’ indoors. Unfortunately, data on indoor air pollutant concentrations are not included in the Albania DHS dataset. Hence, this specific question could be the subject of further research in future studies.

## Figures and Tables

**Table 1:** Summary of Sample Characteristics of Survey Data in Albania.

			Normotensive	Hypertensive	*p*-Value
	Number	Percentage	Percentage	Percentage	
			%	%	
Sex					0.000
Male	5988	28.72	68.6	31.4	
Female	14,858	71.28	70.7	29.2	
Education					0.000
No education	190	0.91	70.5	29.5	
Primary	9593	46.04	64.0	36.1	
Secondary+	11,054	53.05	75.6	24.4	
Wealth					0.000
Poorest	5828	27.96	66.7	33.2	
Poorer	5027	24.11	68.7	31.2	
Middle	4075	19.55	71.04	28.9	
Richer	3526	16.91	72.77	27.2	
Richest	2390	11.47	75.6	24.3	
Not Working					0.000
Working	8515	40.8	27.9	12.93	
Not working	12,331	59.1	71.35	16.9	
BMI					0.000
Underweight	503	7.4	91.55	7.35	
Normal weight	8252	40.3	84.01	15.98	
Overweight	7166	35.0	66.32	33.67	
Obese	4519	22.10	47.9	52.02	
Place of Residence					0.000
Rural	11,303	54.2	68.34	31.65	
Urban	9543	45.7	72.2	27.77	
Age of Households					0.000
15 to 24	4511	21.6	93.48	6.51	
25 to 34	4082	19.58	87.0	13.0	
35 to 44	4091	19.62	73.5	26.5	
45 to 54	5257	25.2	53.2	46.7	
55 to 64	2905	13.93	36.0	63.9	

**Table 2:** Unadjusted and adjusted odds ratios of the association between high blood pressure, indoor air pollution, and selected factors.

	Unadjusted	Adjusted Association
	OR (95% CI)	OR (95% CI)
Household air pollution		
Clean vs. unclean	1.17 (1.10 to 1.24)	1.09 (1.01 to 1.19)
Sex		
Female vs. male	0.90 (0.84 to 0.96)	0.80 (0.74 to 0.86)
Education		
No education	1.29 (0.94 to 1.77)	1.20 (0.84 to 1.71)
Primary	1.75 (1.65 to 1.86)	1.21 (1.12 to 1.30)
Secondary+	1 (reference)	1 (reference)
Wealth		
Poorest	1.54 (1.39 to 1.72)	1.51 (1.29 to 1.76)
Poorer	1.41 (1.26 to 1.58)	1.33 (1.16 to 1.53)
Middle	1.27 (1.13 to 1.42)	1.21 (1.06 to 1.39)
Richer	1.16 (1.03 to 1.31)	1.13 (0.99 to 1.31)
Richest	1 (reference)	1 (reference)
Not Working		
Not working vs. working	0.87 (0.82 to 0.92)	1.02 (0.95 to 1.10)
BMI		
Underweight	0.42 (0.30 to 0.59)	0.84 (0.59 to 1.21)
Normal weight	1 (reference)	1 (reference)
Overweight	2.77 (2.47 to 2.88)	1.50 (1.38 to 1.64)
Obese	5.70 (5.24 to 6.19)	2.67 (2.42 to 2.93)
Place of Residence		
Rural vs. urban	1.20 (1.13 to 1.28)	1.07 (0.98 to 1.16)
Age of Households		
15 to 24	1 (reference)	1 (reference)
25 to 34	2.15 (1.86 to 2.50)	1.78 (1.52 to 2.09)
35 to 44	5.17 (4.5 to 5.93)	3.72 (3.20 to 4.32)
45 to 54	12.60 (11.07 to 14.36)	8.40 (7.27 to 9.69)
55 to 64	25.49 (22.15 to 29.33)	16.86 (14.47 to 19.64)

## Data Availability

All methods were carried out in accordance with relevant guidelines and regulations, and data were sourced from the link below. The data supporting this article are available at: http://dhsprogram.com/data/available-datasets.cfm (accessed on 16 June 2021).
